# Impaired odor recognition memory in Parkinson’s disease linked to absent functional hippocampal asymmetry

**DOI:** 10.1038/s41531-025-00906-3

**Published:** 2025-03-23

**Authors:** Tom Eek, Thomas A. W. Bolton, Nil Dizdar, Maria Larsson, Fredrik Lundin, Charalampos Georgiopoulos

**Affiliations:** 1 Departement of Neurology and Department of Biomedical and Clinical Sciences, Linköping University, Linköping, Sweden; 2 Center for Medical Image and Visualization, Linköping University, Linköping, Sweden; 3 Connectomics Laboratory, Department of Radiology, Centre Hospitalier Universitaire Vaudois (CHUV), Lausanne, Switzerland; 4 Gösta Ekman Laboratories, Department of Psychology, Stockholm University, Stockholm, Sweden; 5 Diagnostic Radiology, Department of Clinical Sciences, Medical Faculty, Lund University, Lund, Sweden

**Keywords:** Parkinson's disease, Predictive markers

## Abstract

Odor recognition memory (ORM) combines olfaction and episodic memory, both linked to dementia and impaired in Parkinson’s Disease (PD). Measuring ORM may indicate early PD dementia and aid in selecting device-aided Parkinson therapy. This study investigates ORM capacity and hippocampal dynamic functional connectivity in PD. Thirty-one PD participants and 31 healthy controls (HC) underwent functional MRI during an ORM task. Co-activation pattern analysis identified active hippocampal networks. The PD group showed impaired ORM and a sequence of four activated hippocampal networks. The fourth network, involving the dorsal Attention Network (dAN), had fewer and shorter expressions during correct ORM responses in PD compared with HC. Hippocampal functional asymmetry was observed in HC but not in PD. These findings suggest that impaired ORM in PD is linked to reduced hippocampal functional asymmetry. Future research should explore differences in functional dynamics of odor memory-related brain regions in PD patients with and without cognitive decline.

## Introduction

Parkinson’s disease (PD) is well-known for its debilitating motor symptoms while non-motor symptoms are poorly understood^1^. Two common non-motor symptoms in PD are olfactory dysfunction and memory deficit. Previous studies demonstrated that approximately 90% of PD patients suffer from olfactory dysfunction and 80% will eventually develop Parkinson’s disease dementia (PDD)^1,2^. Both impairments have been related to α-synuclein pathology onset, for olfaction in the olfactory bulb and anterior olfactory nucleus, and for memory in the hippocampus and other frontotemporal regions^3,4^. Individuals with impaired olfaction reports difficulties in daily life regarding cooking and enjoying food, maintaining personal hygiene and social relationships, as well as experiencing personal safety^5^. Declined cognition and memory in PD have been related to reduced quality of life, and increased distress among patients and their caregivers, playing a key role in the decision concerning nursing home placement^6–8^. In addition to the disease-related cognitive deficit, up to 20% of PD patients demonstrates further cognitive deterioration following device-aided Parkinson therapy (DAPT) such as deep brain stimulation of the subthalamic nucleus (STN-DBS)^9^. Given the profound impact of these non-motor symptoms on daily life, the importance of exploring new methods for early detection of PD patients at risk for developing PDD is clear. This is particularly critical during the “on-off phenomenon” of the disease, when peroral medication is no longer sufficient, and a suitable DAPT should be selected for further symptom relief.

While solid relation between olfactory dysfunction and impaired memory has been demonstrated in numerous studies, particularly related to Alzheimer’s disease (AD), most of the studies have utilized an odor identification test, which mainly draws on semantic memory^10–12^. Semantic memory is defined as the ability to recall facts about the world. According to a recent meta-analysis, odor identification tests also draw on episodic memory^13^. Some types of dementia, such as the dementia seen in AD, are primarily associated with deficits in episodic memory, which refers to the ability to remember past events and contextualize them in space and time^14^. The creation of episodic memories can be divided into three information processing stages: encoding, storage and retrieval. A subcomponent of the retrieval stage is recognition, commonly referring to the ability of recognizing previously encoded stimuli from a set of distractors^15^. A validated odor recognition memory (ORM) test is Sniffin’ TOM which offers a measure of both olfaction and episodic memory^14,16,17^. Given the well-documented deterioration of olfaction and episodic memory as part of the PD course, Sniffin’ TOM may be a sensitive tool for early detection of PDD^2,14,15^.

The hippocampus has a dominant role in cognitive functions that intertwine olfaction with episodic memory, e.g., in successful ORM^18^. Moreover, previous studies have demonstrated a relation between the progression of hippocampal atrophy in PD and the intensification of episodic memory deficit^15,19^. Although the hippocampus is a central brain region in this context, ORM is an associative process that recruits several brain regions. During ORM the hippocampus may also reorganize its interactions with other brain regions over time, adapting to changing memory stages and demands^20,21^. The Toolbox for Co-activation Pattern Analysis (TbCAPs) is a novel method for assessing dynamic functional connectivity (dFC), capturing dynamic hippocampal reconfigurations in functional MRI (fMRI) data. It disentangles networks that interact in a coordinated and sequential manner with a predetermined seed region over time at a frame-wise temporal resolution level^22–24^.

To our knowledge this is the first study with the objective to investigate the capacity for recognizing previously encoded everyday odors and to explore related differences in hippocampal dFC in PD sample eligible for assessment prior STN-DBS, and for whom the early detection of PDD is of great importance.

## Methods

### Participants

Thirty-nine participants with a diagnosis of idiopathic PD according to the UK Brain Bank criteria were recruited from the department of Neurology at Linköping University Hospital^25^. All PD participants experienced ‘on-off phenomena’, characterized by a decline in symptomatic relief during the dosing cycle, and therefore were eligible for assessment prior STN-DBS^26^. Thirty-three non-smoking age-matched healthy controls (HC) reporting good physical and mental health were recruited through advertisements. PD participants were examined by a movement disorder specialist controlling for neurological disorders other than idiopathic PD, psychiatric diagnosis, and evident cognitive, balance, swallowing or speech difficulties. Participants in both groups underwent a passive smelling assessment where they were instructed to judge if they could perceive two different everyday odors.

**Fig. 1:**
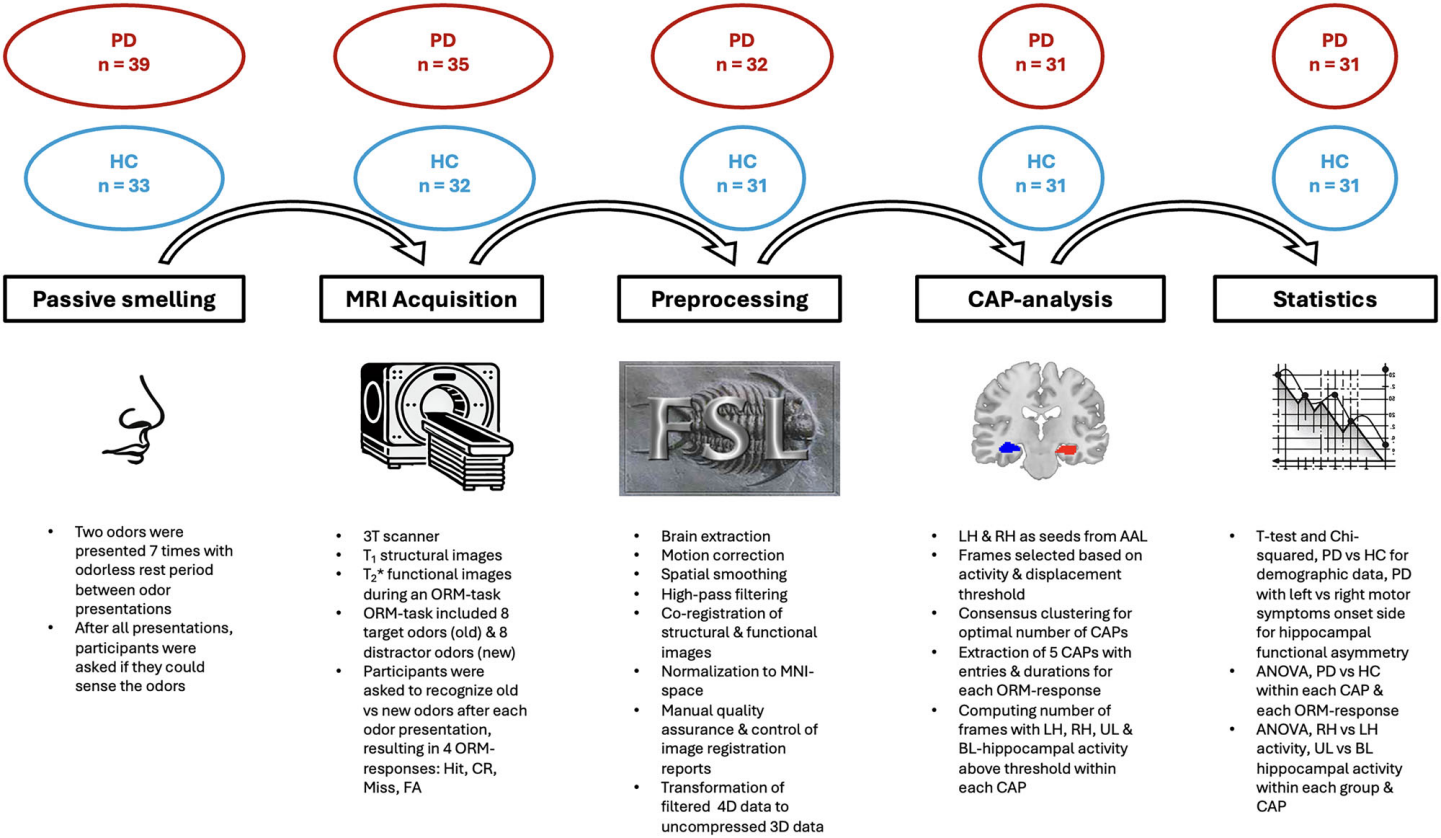
Overview of the different methodological steps in the study and the number of dropouts at each step. PD Parkinson’s Disease, HC healthy controls, ORM odor recognition memory, CR correct rejection, FA false alarm, MNI Montreal Neurological Institute, LH left hippocampus, RH right hippocampus, AAL automated anatomical labeling atlas, CAP co-activation pattern, UL unilateral hippocampal activity, BL bilateral hippocampal activity.

Other exclusion criteria aside from those evaluated in the neurological examination, included reported active colds, allergies or COVID-19 infection, previous nasal cavity surgery, mandibular or magnetic/electromagnetic implants, smoking and loss of sense of smell, meaning no odors were perceived during the passive smelling assessment. Due to loss of sense of smell, substantial movement artifacts, uncompleted fMRI-session or active cold/COVID-19 infection, 8 participants dropped out from the PD group, leaving 31 participants, and 2 from the HC group, leaving 31 participants, refer to Fig. 1 for an overview of the number of dropouts at each methodological step in the study. All participants provided informed consent, and the study was approved by the Swedish Ethical Review Authority (registration number 2019-02679). Participants demographic data, ORM performance and percentage remained frames for analysis are presented in Table 1.

**Table 1:** Demographic data, odor recognition memory performance and percentage remained frames for analysis

	PD (*n* = 31, SD)	HC (*n* = 31, SD)	*P* value
Males/Females	16/15	13/18	*p* = 0.44
Age (years)	59.42 ( ± 8.22)	60.32 ( ± 6.91)	*p* = 0.64
Left/right handedness	4/27	—	—
PD duration (years)	8.55 ( ± 3.25)	—	—
LEDD (mg/day)	1021 ( ± 308.67)	—	—
Hit (proportion)	0.47 ( ± 0.29)	0.72 ( ± 0.15)	*p* < 0.001*
Miss (proportion)	0.53 ( ± 0.29)	0.28 ( ± 0.15)	*p* < 0.001*
CR (proportion)	0.56 ( ± 0.23)	0.62 ( ± 0.14)	*p* = 0.18
FA (proportion)	0.44 ( ± 0.23)	0.38 ( ± 0.14)	*p* = 0.18
Frames remained (%)	40.58 ( ± 6.12)	41.50 ( ± 3.29)	*p* = 0.46

*PD* Parkinson’s Disease, *HC* healthy controls, *SD* standard deviation, *LEDD* levodopa equivalent daily dose, *CR* correct rejection, *FA* false alarm, * significant difference.

### Odor Recognition Memory Task

Building on a prior study the participants were subjected to a trimodal olfactory assessment within the MR-scanner: passive smelling, odor encoding and ORM. This study focuses only on the ORM session which was designed based on Sniffin’ TOM^16^. It included 16 everyday odors: 8 target odors (presented previously during the odor encoding session and therefore labeled as “old”) and 8 distractor odors (presented for the first time during ORM session and therefore labeled as “new”). Target odors were orange, banana, leather, cinnamon, turpentine, lemon, licorice, and garlic. Distractor odors were pineapple, rose, apple, anise, fish, coffee, clove, and peppermint (Burghart Messtechink GmbH, Wedel, Germany). For each odor stimulation, participants were asked to decide whether the odor was “old” or “new” by finger tapping (index = “old” and middle finger = “new”). The answers generated four ORM-responses: Hit (“old” odor correctly recognized as “old”), Miss (“old” odor incorrectly recognized as “new”), Correct Rejection (CR; “new” odor correctly recognized as “new”) and False Alarm (FA; “new” odor incorrectly recognized as “old”). Odor stimulation length was 5 s, followed by 28 s of odorless air. The response window started 3 s after odor stimulation and continued 7 s during the odorless air period. Participants were informed through goggles regarding the different segments of the session (odor stimulation, response and odorless air period). All participants were asked to breath normally through their nose, avoiding sniffing. Odors were delivered simultaneously to both nostrils via Teflon-tubing (4 mm inner diameter) and by OG001 Multistimulator (Burghart Messtechink GmbH, Wedel, Germany), attached to a medical air stream (2.5 L airflow per nostril). Residual odorants were removed by constant inverse airflow within the scanner.

### MRI Acquisition

MRI was conducted with a 3 T scanner (Siemens MAGNETOM Prisma, Siemens AG, Erlangen, Germany) using a 64-channel head-neck coil. Prior to fMRI, a high-resolution, structural T_1_-weighted, 3D volume acquisition was performed. The structural images were used in later co-registration with functional T_2*_-weighted images with blood oxygenation level-dependent (BOLD) contrast. We used a multiplex echo planar imaging (EPI) sequence, containing an initial fat saturation pulse: repetition time [TR] = 878 ms and echo time [TE] = 24 ms; flip angle = 56°; integrated parallel acquisition technic = 3; EPI factor = 68; field of view = 204 × 204 mm^2^; # slices = 45; slice thickness (gap) = 3 (0) mm; voxel = 3 × 3 × 3 mm^3^. Each ORM session generated 620 frames as data points.

### Image Processing

For the preprocessing of the fMRI data, the FMRIB Software Library (FSL) version 6 was used. The data was converted to the standard file format for FSL. For each participants’ structural image, non-brain tissue was removed. Motion estimates during the fMRI-scan were calculated and linear transformations were performed for 3 translation and 3 rotation head motion parameters. On functional images, we applied spatial smoothing at 5 mm full width half maximum Gaussian kernel as well as high-pass temporal filtering. Thereafter, a co-registration of corrected functional images to structural high-resolution images based on brain boundary registration methodology was performed. For normalization, structural and functional images were wrapped non-linearly to the Montreal Neurological Institute (MNI) space. Quality regarding motion estimates and registered images were controlled manually by neuroradiologist for each participant. Poor registration results and absolute head motion greater than 3 mm or relative head motion greater than 1.5 mm entailed exclusion from the study. Additionally based on a movement distortion threshold, images with displacement level higher than 0.5 mm were automatically excluded during the co-activation pattern (CAP) analysis.

### Co-activation Pattern Analysis

Previous studies have demonstrated the central role of the hippocampi both in olfaction and episodic memory^15,18,19^. Hence, the right and the left hippocampus were extracted from the Automated Anatomical Labeling (AAL) atlas and used as seeds in the CAP analysis^22,27^. Selection of frames for analysis was based on a hippocampal activity threshold (either one of the two seeds or both demonstrated an activity level above 0.4) as well as movement distortion level (less than 0.5 mm framewise displacement)^28^.

To determine the optimal number of clusters into which to decompose the retained data, consensus clustering was applied^29^. A candidate range of K = 1 to 10 was explored. In each case, the data from the whole sample (PD + HC) was repeatedly subsampled (100%, 15 iterations) and clustered to yield a consensus matrix. Consensus values equal to 0/1 highlight pairs of frames that are respectively never/always clustered together, denoting stability. The percentage of ambiguously clustered pairs of frames (PAC; for which consensus took intermediate values) was used as a metric indicative of clustering quality and resulted in an optimum number of clusters, hence the optimal number of CAPs (smallest PAC)^30^. For each ORM scan, time points were labeled as reflective of either a CAP or baseline hippocampal configuration. This labeling was based on whether one or both seeds demonstrated an activity level above the threshold, in conjunction with simultaneous strong and consistent activity in other brain areas. The term ‘expression’ is used throughout the text to denote above threshold activity both regarding hippocampal activations and CAPs.

Next, we focused on the epochs of odor stimulation and ORM-response (15 time points, 1 time point = 878 ms). For each CAP, we computed entries (number of times a given CAP was entered) and duration (the mean time in sec a CAP was expressed). The same procedure was performed in relation to baseline when no task was performed. Furthermore, for the examination of hippocampus lateralization within each CAP the number of frames associated with both hippocampi, left hippocampus and right hippocampus were extracted for each group.

### Statistics

We performed a t-test and Chi-squared test to examine if the PD sample differed from HC regarding demographic data, ORM performance and remained frames for analysis, refer to Table 1. Thereafter, an ANOVA was conducted to examine differences between samples regarding Baseline entries and duration and CAP entries and duration related to each ORM-response. The results of the ANOVA for Baseline Configuration and Network 4 related to each ORM-response are presented in Table 2, for results regarding all the networks refer to Supplementary Table 1. For the examination of hippocampus lateralization and dynamics, we computed a unilateral activation value by adding only left hippocampus to only right hippocampus expressions for each group. This value was compared within each group to a bilateral activation value (right and left hippocampus expressed simultaneously) in a paired t-test. We compared as well only right versus only left hippocampus expressions, see Table 3. Finally, we divided the PD group based on the side of motor symptoms onset and computed an asymmetry value (mean left hippocampus expressions - right hippocampus expressions) for each network. A post-hoc paired t-test was conducted to investigate if previous results concerning hippocampus lateralization were related to motor symptoms onset side, refer to Table 4. All comparisons were Bonferroni-corrected. To further examine the relationship between asymmetric hippocampal activity (AHA) and ORM performance in PD, we divided the PD sample into two subsamples for each network: PD with and without AHA. This categorization was based on AHA values within and outside the 95% confidence interval. We then performed a post-hoc t-test, comparing the ORM performance of the two subsamples.

**Table 2:** Comparison between PD and HC related to hippocampal dynamic functional connectivity for the Baseline Configuration and for ORM-responses within Network 4

Network dynamics	ORM response	PD (SD)	HC (SD)	*P* value corr.
Baseline entries	−	81.38 ( ± 23.95)	65.25 ( ± 15.48)	*p* = 0.06
Baseline duration (sec)	−	4.18 ( ± 1.63)	5.21 ( ± 1.87)	*p* = 0.50
Network 4 entries	Hit	1.27 ( ± 0.59)	1.64 ( ± 0.48)	*p* = 0.24
	Miss	1.54 ( ± 0.71)	1.91 ( ± 0.79)	*p* = 1.00
	CR	1.39 ( ± 0.63)	1.95 ( ± 0.64)	*p* = 0.02*
	FA	1.60 ( ± 0.71)	1.89 ( ± 0.70)	*p* = 1.00
Network 4 duration (sec)	Hit	1.39 ( ± 0.63)	1.92 ( ± 0.69)	*p* = 0.08
	Miss	1.50 ( ± 0.61)	1.93 ( ± 0.77)	*p* = 0.54
	CR	1.46 ( ± 0.61)	1.85 ( ± 0.69)	*p* = 0.56
	FA	1.37 ( ± 0.57)	1.78 ( ± 0.81)	*p* = 0.54

*ORM* odor recognition memory, *PD* Parkinson’s disease, *HC* healthy controls, *CR* correct rejection, *FA* false alarm, *SD* standard deviation, *corr*. Bonferroni-corrected (5 networks × 2 samples × 2 independent ORM responses = 20); *, significant difference.

**Table 3:** Comparisons within each group regarding the mean number of bilateral vs unilateral hippocampus expressions and left versus right hippocampus expressions

	Network	Bilateral hippocampus (SD)	Unilateral hippocampus (SD)	*P* value corr.	Left hippocampus (SD)	Right hippocampus (SD)	*P* value corr.
PD	**Network 1**	20.03 ( ± 8.42)	19.12 ( ± 8.35)	*p* = 1.00	9.64 ( ± 5.45)	9.48 ( ± 6.38)	*p* = 1.00
	**Network 2**	32.38 ( ± 14.22)	30.74 ( ± 14.13)	*p* = 1.00	18.83 ( ± 8.28)	16.90 ( ± 8.52)	*p* = 0.71
	**Network 3**	24.80 ( ± 13.90)	15.00 ( ± 8.11)	*p* = 0.03*	6.87 ( ± 6.02)	8.12 ( ± 6.08)	*p* = 1.00
	**Network 4**	36.35 ( ± 15.49)	31.41 ( ± 11.14)	*p* = 1.00	16.93 ( ± 7.31)	14.48 ( ± 6.73)	*p* = 1.00
	**Network 5**	23.80 ( ± 11.17)	17.96 ( ± 7.50)	*p* = 0.17	8.58 ( ± 5.66)	9.38 ( ± 4.46)	*p* = 1.00
HC	**Network 1**	18.35 ( ± 9.14)	16.67 ( ± 7.28)	*p* = 1.00	10.22 ( ± 5.20)	6.45 ( ± 3.91)	*p* = 0.01*
	**Network 2**	29.32 ( ± 10.04)	30.80 ( ± 11.69)	*p* = 1.00	10.96 ( ± 5.72)	19.83 ( ± 9.31)	*p* < 0.01*
	**Network 3**	29.64 ( ± 13.24)	10.09 ( ± 5.78)	*p* < 0.01*	4.29 ( ± 3.40)	5.80 ( ± 3.92)	p = 0.73
	**Network 4**	51.83 ( ± 19.57)	30.35 ( ± 10.59)	*p* < 0.01*	17.77 ( ± 7.66)	12.58 ( ± 5.87)	*p* = 0.02*
	**Network 5**	24.70 ( ± 9 0.23)	15.54 ( ± 4.71)	*p* < 0.01*	7.48 ( ± 3.52)	8.06 ( ± 3.45)	*p* = 1.00

*PD* Parkinson’s disease, *HC* healthy controls, *SD* standard deviation, *corr.* Bonferroni-corrected (5 networks × 2 samples = 10); *, significant difference.

**Table 4:** Comparison regarding hippocampal asymmetry value between PD with left vs right motor symptom onset (Positive values indicate higher number of left hippocampus expressions compared with right hippocampus expressions, while negative values indicate the opposite relation)

Network	PD left motor symptoms onset (*n* = 18, SD)	PD right motor symptoms onset (*n* = 13, SD)	*P* value corr.
Network 1	3.11 ( ± 4.37)	−3.92 ( ± 10.95)	*p* = 0.22
Network 2	−2.61 ( ± 9.22)	−3.69 ( ± 9.27)	*p* = 1.00
Network 3	−5.66 ( ± 6.87)	4.84 ( ± 8.10)	*p* < 0.005*
Network 4	4.83 ( ± 7.31)	−0.84 ( ± 9.37)	*p* = 0.41
Network 5	−0.05 ( ± 6.26)	−1.84 ( ± 7.88)	*p* = 1.00

*PD* Parkinson’s disease, *SD* standard deviation, *corr.* Bonferroni-corrected (5 networks × 2 samples = 10); *, significant difference.

## Results

### Samples Characteristics

Our results suggested that the PD sample suffered from “on-off phenomena” and was eligible for assessment prior STN-DBS considering PD participants mean age ($\bar{x}=$ 59.42 ± 8.22 years), mean levodopa equivalent daily dose ($\bar{x}=$ 1021 ± 308.67 mg) and mean PD duration since motor symptoms onset ($\bar{x}$ = 8.55 ± 3.25 years). Approximately 12% of the PD participants confirmed left-handedness. The PD sample did not differ from the HC regarding sex distribution, age, percentage frames retained for analysis, CR and FA proportion. Nevertheless, the PD sample compared with HC demonstrated lower Hit and higher Miss proportions (*p* < 0.001), refer to Table 1 for more information.

### Hippocampal Co-activation Patterns

After selection of frames based on hippocampal activity threshold and movement distortion level, more than 40% of the frames in both groups retained for analysis, for more information see Table 1. Consensus clustering generated an optimum number of 5 clusters, hence 5 hippocampal CAPs (networks).

**Fig. 2:**
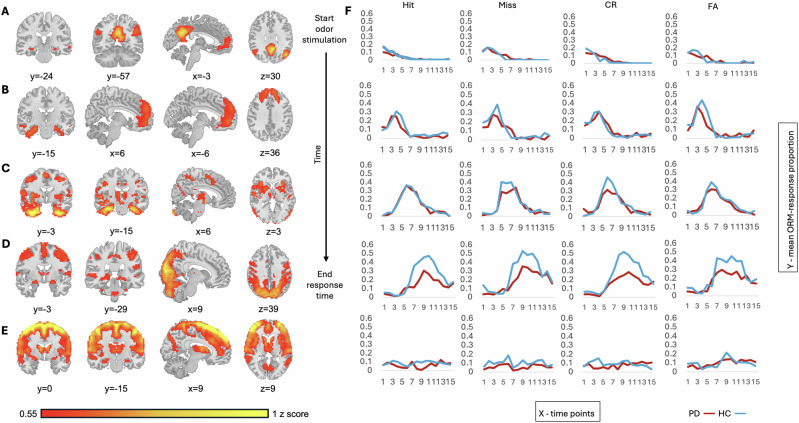
Illustration of the following hippocampal networks. **A** Network 1 incorporating parts of the posterior default mode network (posterior cingulate gyrus, precuneus, angular gyrus). **B** Network 2 incorporating parts of the anterior default mode network (paracingulate gyrus, anterior cingulate gyrus, and superior frontal gyrus). **C** Network 3 incorporating parts of the salience network (insula and dorsal cingulate gyrus). **D** Network 4 incorporating parts of the dorsal attention network (middle frontal gyrus and brain regions along the intraparietal sulcus), visual cortex, cerebellum, and thalamus. **E** Network 5 incorporating parts of the ventral attention network (interior frontal gyrus and frontal operculum). **F** Mean odor recognition memory (ORM) response proportion for each response: Hit, Miss, CR; correct rejection and FA; false alarm, over time during 15 time points (1 time point = 878 ms, total time 13.17 s) and related to each parallel presented network. The recruited networks (aside from Network 5 which did not demonstrate clear sequential order) as well as the odor recognition memory responses are ordered on a vertical chronological timeline.

Four networks demonstrated a chronological dFC and are presented in their sequential order. Network 1 (peak 1 - 2 sec after odor stimulation) incorporated parts of the posterior default mode network (DMN; posterior cingulate gyrus, precuneus, angular gyrus). Network 2 (peak 3–4 s after odor stimulation) incorporated the parahippocampal gyrus as well as parts of the anterior DMN (paracingulate gyrus, anterior cingulate gyrus, and superior frontal gyrus). Network 3 (peak 5–6 sec after odor stimulation) incorporated parts of the olfactory cortex (amygdala and entorhinal cortex) and its projections (thalamus and basal ganglia), supplementary motor area and parts of the salience network (SN; insula and dorsal cingulate gyrus). Network 4 (peak 7–10 s after odor stimulation) incorporated the hippocampus (mostly dentate gyrus), the thalamus, the visual cortex, the cerebellum, and parts of the dorsal attention network (AN; middle frontal gyrus and brain regions along the intraparietal sulcus). Network 5 which did not have a clear position in this sequential order incorporated the superior temporal gyrus, the cingulate gyrus, the supplementary motor area, the precentral gyrus, the postcentral gyrus, the precuneus, the thalamus and parts of the ventral AN (interior frontal gyrus and frontal operculum), see Fig. 2.

The ANOVA comparing the two samples regarding the two dFC parameters: entries and duration, generated significant results only within the Baseline Configuration and Network 4, for results regarding all the networks refer to Supplementary Table 1. While not significant, the PD participants compared with HC tended to enter the Baseline Configuration more frequently (*p* = 0.06). This indicated that the hippocampus activity level was under the threshold more frequently in PD compared to HC while the mean duration did not differ between the groups (*p* = 0.50). Within Network 4, we observed differences only in relation to correct ORM-responses. The PD group compared to HC entered Network 4 less frequently during CR (*p* = 0.02), no difference was observed during Hit (*p* = 0.24). Nevertheless, PD participants compared to HC showed a tendency to a shorter expression time of Network 4 during Hit (*p* = 0.08), no difference was observed during CR (*p* = 0.56), see Table 2.

### Hippocampal Lateralization

The PD sample demonstrated a difference only within Network 3 where the number of bilateral hippocampus expressions were larger than unilateral (*p* = 0.03). No other differences were found in this group, for more information see to Table 3. To investigate if these results were related to motor symptoms predominance, we compared post-hoc between PD participants with left versus right motor symptoms onset side regarding asymmetry within each network, for more information refer to Table 4. No differences were found aside from within Network 3. PD participants with left compared with right motor symptoms onset side demonstrated more expressions of right versus left hippocampus and higher asymmetry level (*p* < 0.005), indicating that only Network 3 was dependent on motor symptoms.

To further investigate the hypothesis that asymmetric hippocampal activity (AHA) in PD is related to ORM performance rather than to the side of motor symptoms onset, we divided the PD sample into two subsamples: PD with and without AHA. In a post-hoc comparison analysis, a significant difference between the subsamples was observed in CR and FA only within Network 2 (*p* < 0.05). Specifically, PD participants with AHA demonstrated higher CR and lower FA proportion compared to those without AHA, for more details refer to Supplementary Table 2.

Conversely the HC group demonstrated higher number of bilateral hippocampus expressions compared to unilateral within several networks: Network 3, Network 4, and Network 5 (*p* < 0.01). Differences were also observed in relation to left versus right hippocampus expressions: Network 1 (number of left expressions was higher than right, *p* = 0.01) and Network 2 (number of right expressions was higher than left, *p* < 0.01). For Network 4 which also demonstrated higher bilateral hippocampus expressions compared to unilateral (*p* < 0.01), the number of left hippocampus expressions was higher than right (*p* = 0.02).

## Discussion

In this cross-sectional study, we investigated differences between PD patients eligible for assessment prior STN-DBS and HC, regarding ORM and its related hippocampal dFC. In accordance with previous studies, our results showed that PD patients suffer from ORM impairment, particularly related to successful odor recognition memory^14,31,32^.

Our CAP-analysis revealed 5 hippocampal networks while 4 demonstrated a chronological and cognitive processing order. Network 1 incorporated parts of the posterior DMN associated with self-centered cognition such as monitoring and representation of the self^33^. Network 2 incorporated parts of the anterior DMN related to planning and decision making^33^. Network 3 incorporated parts of the olfactory cortex, its projections (thalamus and basal ganglia) and the SN, these neuronal structures known to be involved in processing of odors and emotions, preparing and execution of voluntary movements, network shifting, detection of relevant stimulus among multiple inputs and integration of information from various circuits such as sensory, affective and cognitive^20,34,35^. Network 4 incorporated mainly the dentate gyrus of the hippocampus, the visual cortex, the cerebellum, and parts of the dorsal AN, neuronal structures related to pattern separation (transformation of similar experiences into distinct memory traces), associative memory retrieval (memories formed of several modalities such as olfactory, visual etc.), working memory, visual processing, cognition and imagery, sniffing, top-down attentional control and goal-directed behavior^36–38^. Network 5 which did not have a clear position in this temporal sequence incorporated parts of the ventral AN associated with reorienting attention and bottom-up attentional control^38,39^.

When the PD sample was compared with HC some differences and tendencies were observed in the Baseline Configuration and Network 4. PD participants tended to remain more frequently in the Baseline Configuration, meaning that the hippocampi were activated more times but at lower level than the selected threshold. Although not related to PD, previous studies have demonstrated a relation between memory impairment, risk for developing dementia and increased activity of the hippocampus during rest^40,41^. Within Network 4, indicative results were observed particularly in relation to correct ORM-responses. PD participants showed a tendency to a shorter expression time of Network 4 during Hit which was aligned with their lower performance at recognizing previously encoded odors. The PD sample also demonstrated a lower number of expressions of Network 4 during CR while no difference between the samples was observed in behavioral data. These findings are to some degree aligned with previous studies suggesting a common hippocampal component for Hit and CR while our results differ regarding the direction of the relation (positive instead of negative direction)^20,42^. Furthermore, lower activity in the dorsal AN during rest (part of Network 4) has been related to mild cognitive impairment in PD^43^.

Nevertheless, Network 4 is a broad CAP and it is unclear which brain regions and consequently functions drive the differences between the samples. Included in this network were the cerebellum and the visual cortex. The cerebellum is involved in sniffing and respiratory regulation which are affected in PD^44^. The visual cortex is associated with visual processing, cognition and imagery which are also impaired in PD^45^. Yet, both brain structures have demonstrated involvement in odor memory formation^20^. Moreover, it is more likely that the differences are related to successful ORM since the participants were instructed not to sniff and the observed differences were particularly in relation to Hit and CR and not to other ORM-responses.

Other differences between the samples were revealed in the relation between the hippocampi within each network. Mostly, PD participants did not demonstrate differences regarding left vs right and bilateral vs unilateral hippocampus expressions, aside from within Network 3. Although during rest, lack of hippocampal functional asymmetry has been observed in Alzheimer’s disease^46^. Within Network 3, bilateral expressions occurred more frequently than unilateral. Since this network included the basal ganglia and a difference in hippocampal functional asymmetry between PD participants with left vs right motor symptoms predominance, it is likely to conclude that this difference is related to motor symptoms. To further investigate the hypothesis that asymmetric hippocampal activity (AHA) in PD is related to ORM performance rather than to the side of motor symptoms onset, we performed a post-hoc comparison between PD participants with and without AHA. Preliminary findings suggest that PD participants with AHA in Network 2 have higher CR and lower FA proportion compared to those without AHA. However, these results should be interpreted with caution due to the small size of the subsamples and should serve as a basis for future research. Conversely, HC showed a functional asymmetry within all networks either in the comparison of bilateral vs unilateral or left vs right hippocampus expressions. Only within Network 4, HC demonstrated both more frequent bilateral and left hippocampus expressions. Although not related to the hippocampus, previous studies demonstrated right lateralization of olfaction associated with odor memory^42^. This contradiction may be related to a more complex approach regarding lateralization in this study where it is analyzed in relation to each memory phase and therefore to each recruited network during the process of ORM.

Our study has some limitations that should be addressed. The samples were not screened for cognitive status or biomarkers, which may put in question the results regarding ORM performance and the fMRI, as these factors could affect both. Nevertheless, PD participants underwent a neurological assessment by a specialist in movement disorders without remarks and HC showed a normal ORM performance, giving some indication of intact cognitive status. The samples could not be compared regarding handedness distribution since this information was not available for HC. Nevertheless, our PD sample demonstrated a similar handedness distribution to the normal population, with approximately 10% being left-handed^47^. PD participants were not screened in relation to their disease stage with validated scales which might led to a heterogeneous PD sample and therefore distorted findings. However, all PD participants were evaluated to be at the start of their “on-off phenomena” which was in accordance with the data regarding LEDD and PD duration since first experienced symptoms. Dopaminergic medication in PD could also influence our results since it has been shown to increase brain activity, e.g., of the posterior DMN^33^. However, in general our study indicated the opposite direction, meaning decreased brain activity in PD. Finally, the delivery of odors was not synchronized with the participants’ respiratory cycles, as such measurements were not conducted in our study. This could affect the interpretation of our findings. However, the participants were instructed to breath normally and not to sniff the odors.

This study paves the way for new clinical implications. Our findings of significant differences in hippocampal functional dynamic connectivity during the ORM task between PD and HC may aid in early detection of mild cognitive impairment and dementia in PD, thereby improving DAPT selection. To examine the clinical value of the ORM task, future research should focus on the functional dynamics of odor memory-related brain regions in larger PD samples with and without cognitive decline.

## Supplementary information

Supplementary material

## Data Availability

The data supporting our findings is available from the corresponding author, upon reasonable request.
