# Supplementary material for: Impaired odor recognition memory in Parkinson’s disease linked to absent functional hippocampal asymmetry
Source: NPJ Parkinsons Dis. 2025 Mar 23;11:56. doi: 10.1038/s41531-025-00906-3 (PMC11930930; doi:10.1038/s41531-025-00906-3)
Supplement: Supplementary file 1 — Supplementary material [file 41531_2025_906_MOESM1_ESM.docx]

**Supplementary material**

**Supplementary Table 1.** Comparison between PD and HC related to hippocampal dynamic functional connectivity

or the Baseline Configuration and for ORM-responses within each Network.

| Network dynamics | ORM response | PD (SD) | HC (SD) | P value corr. |
| --- | --- | --- | --- | --- |
| Baseline entries | - | 81.38 (± 23.95) | 65.25 (± 15.48) | p = .06 |
| Baseline  duration (sec) | - | 4.18 (± 1.63) | 5.21 (± 1.87) | p = .50 |
| Network 1 entries | Hit | .33 (± .40) | .41 (± .28) | p = 1.00 |
|  | Miss | .47 (± .51) | .31 (± .41) | p = 1.00 |
|  | CR | .48 (± .39) | .43 (± .38) | p = 1.00 |
|  | FA | .45 (± .44) | .47 (± .45) | p = 1.00 |
| Network 1 duration (sec) | Hit | 1.13 (± .29) | 1.16 (± .33) | p = 1.00 |
|  | Miss | 1.16 (± .33) | 1.64 (± .77) | p = 1.00 |
|  | CR | 1.20 (± .62) | 1.15 (± .33) | p = 1.00 |
|  | FA | 1.27 (± .46) | 1.27 (± .56) | p = 1.00 |
| Network 2 entries | Hit | .79 (± .48) | 1.03 (± .47) | p = 1.00 |
|  | Miss | 1.00 (± .65) | 1.26 (± .64) | p = 1.00 |
|  | CR | 1.15 (± .60) | 1.01 (± .45) | p = 1.00 |
|  | FA | 1.00 (± .62) | 1.21 (± .57) | p = 1.00 |
| Network 2 duration (sec) | Hit | 1.19 (± .40) | 1.25 (± .37) | p = 1.00 |
|  | Miss | 1.31 (± .55) | 1.25 (± .52) | p = 1.00 |
|  | CR | 1.20 (± .33) | 1.33 (± .42) | p = 1.00 |
|  | FA | 1.37 (± .61) | 1.39 (± .55) | p = 1.00 |
| Network 3 entries | Hit | 1.13 (± .65) | 1.08 (± .46) | p = 1.00 |
|  | Miss | 1.00 (± .42) | 1.22 (± .60) | p = 1.00 |
|  | CR | 1.03 (± .63) | 1.17 (± .51) | p = 1.00 |
|  | FA | 1.06 (± .52) | 1.17 (± .49) | p = 1.00 |
| Network 3 duration (sec) | Hit | 1.53 (± 1.07) | 1.68 (± .96) | p = 1.00 |
|  | Miss | 1.61 (± .82) | 1.60 (± .60) | p = 1.00 |
|  | CR | 1.62 (± .77) | 1.75 (± .60) | p = 1.00 |
|  | FA | 1.54 (± .94) | 1.61 (± 1.02) | p = 1.00 |
| Network 4 entries | Hit | 1.27 (± .59) | 1.64 (± .48) | p = .24 |
|  | Miss | 1.54 (± .71) | 1.91 (± .79) | p = 1.00 |
|  | CR | 1.39 (± .63) | 1.95 (± .64) | p = .02* |
|  | FA | 1.60 (± .71) | 1.89 (± .70) | p = 1.00 |
| Network 4 duration (sec) | Hit | 1.39 (± .63) | 1.92 (± .69) | p = .08 |
|  | Miss | 1.50 (± .61) | 1.93 (± .77) | p = .54 |
|  | CR | 1.46 (± .61) | 1.85 (± .69) | p = .56 |
|  | FA | 1.37 (± .57) | 1.78 (± .81) | p = .54 |
| Network 5 entries | Hit | .68 (± .48) | .84 (± .44) | p = 1.00 |
|  | Miss | .68 (± .46) | 1.12 (± .87) | p = .42 |
|  | CR | .83 (± .49) | .94 (± .51) | p = 1.00 |
|  | FA | .94 (± .89) | .94 (± .46) | p = 1.00 |
| Network 5 duration (sec) | Hit | 1.11 (± .31) | 1.27 (± .38) | p = 1.00 |
|  | Miss | 1.13 (± .41) | 1.12 (± .34) | p = 1.00 |
|  | CR | 1.28 (± .61) | 1.30 (± .31) | p = 1.00 |
|  | FA | 1.35 (± .71) | 1.32 (± .49) | p = 1.00 |

**Abbreviations:** ORM, odor recognition memory; PD, Parkinson’s disease; HC, healthy controls; CR, correct rejection; FA, false alarm; SD, standard deviation; corr., Bonferroni-corrected (5 networks x 2 samples x 2 independent ORM responses = 20); *, significant.

**Supplementary Table 2.** Comparison between PD participants with and without asymmetric hippocampus activity related to each ORM-response proportion and network.

| Network dynamics | ORM response | No asymmetric hippocampal activity (SD) | Asymmetric hippocampal activity (SD) | P value. |
| --- | --- | --- | --- | --- |
| Sample size (n) |  |  |  |  |
| Network 1 | Hit | .50 (±.28) | .38 (±.33) | p = .36 |
| PD no AHA, n = 25  PD AHA, n = 6 | Miss | .50 (±.28) | .62 (±.33) | p = .36 |
|  | CR | .52 (±.24) | .69 (±.15) | p = .14 |
|  | FA | .48 (±.24) | .31 (±.15) | p = .14 |
| Network 2 | Hit | .54 (±.28) | .37 (±.29) | p = .11 |
| PD no AHA, n = 18  PD AHA, n = 13 | Miss | .46 (±.28) | .63 (±.29) | p = .11 |
|  | CR | .48 (±.23) | .65 (±.21) | p = .04* |
|  | FA | .52 (±.23) | .35 (±.21) | p = .04* |
| Network 3 | Hit | .48 (±.28) | .46 (±.33) | p = .86 |
| PD no AHA, n = 22  PD AHA, n = 9 | Miss | .52 (±.28) | .54 (±.33) | p = .86 |
|  | CR | .52 (±.21) | .61 (±.29) | p = .36 |
|  | FA | .48 (±.21) | .39 (±.29) | p = .36 |
| Network 4 | Hit | .45 (±.28) | .56 (±.35) | p = .41 |
| PD no AHA, n = 25  PD AHA, n = 6 | Miss | .55 (±.28) | .44 (±.35) | p = .41 |
|  | CR | .56 (±.24) | .50 (±.23) | p = .54 |
|  | FA | .44 (±.24) | .50 (±.23) | p = .54 |
| Network 5 | Hit | .47 (±.29) | .47 (±.32) | p = .99 |
| PD no AHA, n = 25  PD AHA, n = 6 | Miss | .53 (±.29) | .53 (±.32) | p = .99 |
|  | CR | .54 (±.23) | .56 (±.24) | p = .87 |
|  | FA | .46 (±.23) | .44 (±.24) | p = .87 |

**Abbreviations:** ORM, odor recognition memory; PD, Parkinson’s disease; AHA, asymmetric hippocampal activity, CR, correct rejection; FA, false alarm; SD, standard deviation; n, sample size; *, significant.
